# Review of a two-year methicillin-resistant Staphylococcus aureus screening program and cost-effectiveness analysis in Singapore

**DOI:** 10.1186/s12879-015-1131-5

**Published:** 2015-09-29

**Authors:** Mar-Kyaw Win, Tarek Abdellatif Aly Soliman, Linda Kay Lee, Chia Siong Wong, Angela Chow, Brenda Ang, Carrasco L. Roman, Yee-Sin Leo

**Affiliations:** Institute of Infectious Diseases and Epidemiology, Tan Tock Seng Hospital, Singapore, Singapore; Saw Swee Hock School of Public Health, National University of Singapore, Singapore, Singapore; Department of Biological Sciences, National University of Singapore, Singapore, Singapore

**Keywords:** Methicillin-resistant staphylococcus aureus, Colonization and acquisition, Screening program, Economic analysis

## Abstract

**Background:**

Methicillin-resistant *Staphylococcus aureus* (MRSA) poses an increasingly large disease and economic burden worldwide. The effectiveness of screening programs in the tropics is poorly understood. The aims of this study are: (i) to analyze the factors affecting MRSA colonization at admission and acquisition during hospitalization and (ii) to evaluate the cost-effectiveness of a screening program which aims to control MRSA incidence during hospitalization.

**Methods:**

We conducted a retrospective case–control study of patients admitted to the Communicable Disease Centre (CDC) in Singapore between Jan 2009 and Dec 2010 when there was an ongoing selective screening and isolation program. Risk factors contributing to MRSA colonization on admission and acquisition during hospital stay were evaluated using a logistic regression model. In addition, a cost-effectiveness analysis was conducted to determine the cost per disability-adjusted life year (DALY) averted due to implementing the screening and isolation program.

**Results:**

The average prevalence rate of screened patients at admission and the average acquisition rate at discharge during the study period were 12.1 and 4.8 % respectively. Logistic regression models showed that older age (adjusted odds ratio (OR) 1.03, 95 % CI 1.02–1.04, *p* < 0.001) and dermatological conditions (adjusted OR 1.49, 95 % CI 1.11–1.20, *p* = 0.008) were independently associated with an increased risk of MRSA colonization at admission. Age (adjusted OR 1.02, 95 % CI 1.01–1.03, *p* = 0.002) and length of stay in hospital (adjusted OR 1.04, 95 % CI 1.03–1.06, *p* < 0.001) were independent factors associated with MRSA acquisition during hospitalization. The screening and isolation program reduced the acquisition rate by 1.6 % and was found to be cost saving. For the whole study period, the program cost US$129,916, while it offset hospitalization costs of US$103,869 and loss of productivity costs of US$50,453 with −400 $/DALY averted.

**Discussion:**

This study is the first to our knowledge that evaluates the cost-effectiveness of screeningand isolation of MRSA patients in a tropical country. Another unique feature of the analysis is the evaluationof acquisition rates among specific types of patients (dermatological, HIV and infectious disease patients)and the comparison of the cost-effectiveness of screening and isolation between them.

**Conclusions:**

Overall our results indicate high MRSA prevalence that can be cost effectively reduced by selective screening and isolation programs in Singapore.

## Background

Methicillin-resistant *Staphylococcus aureus* (MRSA) is a significant cause of nosocomial infection. Once acquired from a contaminated source, MRSA can lead to asymptomatic colonization or serious infection. The latter has caused substantial healthcare and economic burden worldwide. For instance, MRSA-related hospitalizations in the United States increased more than twofold from 1997 to 2005 [1] and a recent report revealed that there were over 11,000 MRSA infection related deaths in 2011 http://www.cdc.gov/drugresistance/threat-report-2013/. In the European Union, additional hospital stay due to MRSA infection cost hospitals 380 million euros in 2010 [2].

The World Health Organization (WHO) has published guidelines and recommendations for prevention and control of healthcare associated infections including MRSA http://whqlibdoc.who.int/publications/2011/9789241501507_eng.pdf. Various hospitals and countries have utilized their own practices to suit their needs and resources for hospital control of MRSA. The Netherlands has successfully carried out a “search-and-destroy” policy [3], while the United Kingdom favors targeted screening and control [4]. The screening method and target population are however debatable. Rapid, universal MRSA testing is seemingly ideal but may be expensive and unwarranted [5]; selective screening, while cost-effective, may result in missed cases [6].

Cost-effectiveness studies of MRSA control programs are limited [7, 8]. In USA, it has been shown that screening programs would present a cost of 19 to 27 times less than the cost posed by MRSA [9]. Similar results were found in the Netherlands, Sweden, and France where the net value of screening and preventive isolation programs was shown to be positive [10–12]. In Germany, the cost of treatment and isolation interventions has been shown to be half the cost of MRSA colonization [13]. In the UK, screening and decolonization was shown to be robustly cost-effective to prevalence of MRSA on admission [14]. In Australia, it was shown that peroxide decontamination reduced MRSA acquisition from 9 to 5.3 per 10,000 patient days [15, 16]. Cost-benefit analyses in tropical countries are, however, very rare and it would be useful to obtain estimates of MRSA prevalence, effectiveness and cost-effectiveness of screening programs in the tropics to compare with those in temperate regions.

MRSA is endemic to major hospitals and is among the most important hospital-acquired pathogens in Singapore [17, 18], where serious outbreaks have occurred [19, 20]. Incidence and prevalence rates of MRSA have remained constant over the past two decades in major academic medical centers. In 2006, a laboratory based surveillance program of antimicrobial drug-resistance in six public sector acute-care hospitals in Singapore revealed that 35 % of isolates were MRSA [17]. Among surveyed Western Pacific countries, this proportion of methicillin resistance ranged from 23.6 to 73.8 % [21].

Numerous studies have estimated the prevalence of MRSA at admission to hospitals. In the UK, MRSA colonization in surgical patients was 5.1 and 6.7 % in emergency patients. 5.1 % among all patients at a Swiss teaching hospital [22–24]. There are fewer reports describing the MRSA colonization status of patients admitted to Singapore hospitals. In Singapore, MRSA colonization rate in HIV outpatients and in emergency patients were 3 and 1.8 % respectively, and *Staphylococcus aureus* colonization was 23 % [25, 26].

A case–control study conducted at two hospitals in Singapore found that mortality, length of stay, hospitalization cost, and losses of quality of life were associated with MRSA infection [27]. Some other risk factors such as the presence of co-morbid medical condition and the use of medical devices have been shown to be associated with MRSA infections and colonization [28, 29].

An MRSA screening program for selected inpatients began in January 2009 and is ongoing at the Communicable Disease Centre (CDC) at Tan Tock Seng Hospital (TTSH), which is the primary center for infectious disease management in Singapore; it also historically houses patients with dermatological conditions. TTSH is a 1600-bed adult tertiary-care public hospital with more than 4500 admissions per month during the study period. It is also the national referral center for HIV infection and emerging infectious diseases in Singapore. The program was implemented to determine the MRSA colonization status of incoming patients and the proportion of inpatients who acquire MRSA during CDC hospitalization.

The aims of this study are: (i) to analyze the factors affecting MRSA colonization at admission and acquisition during hospitalization and (ii) to evaluate the cost-effectiveness of the CDC screening program which aims to control MRSA incidence during hospitalization.

## Methods

### Data collection and experimental design

On-going inpatient admissions and discharge screening for MRSA was conducted during the 24 month study period (Jan 2009–Dec 2010) in three wards that housed patients in different categories (1) ward A, HIV (human immunodeficiency virus) and/or other infectious disease (ID) patients (patients admitted for dengue fever, malaria, chicken pox, etc.), (2) ward B, dermatological patients (patients with skin disease) and/or ID patients, and (3) ward C, HIV patients. Only HIV positive patients were screened in ward A while all patients in the other two wards were screened. Ward B is composed of 22 single-bed rooms with attached bathroom and toilet. The other two have a mixture of single and multi-bed rooms. Ward A is structured with 4 single rooms and 12 multi-bed rooms whereas ward C has 9 single rooms with 1 multi-bed room. Each multi-bed room has 2–4 beds.

Upon admission, known MRSA positive patients (previous known MRSA colonization or infection that was laboratory confirmed irrespective of time) were isolated or allocated to cohorts; they were not screened upon entry or exit. Those who tested positive during initial screening were isolated and not retested at discharge. Patients whose MRSA status was negative at entry were screened upon discharge. Three swabs were taken by trained health care workers at five sites—one swab for the nares, axilla, and groin, one for the perianal region, and one for the throat. Any open wounds were swabbed as well. Samples were inoculated on chromogenic agar plates (MRSASelect, BioRad, France) and incubated at 37 °C for 18–28 h. Growth of pink or mauve colonies was interpreted as MRSA positive, while colorless colonies were MRSA negative.

MRSA negative patients hospitalized for more than 24 h were swabbed at the same sites upon discharge. Those who initially tested negative at entry but positive at exit were determined to have acquired MRSA during hospitalization. Any MRSA negative patients who were readmitted to CDC during the study period were re-screened upon entry and exit, if appropriate.

Demographic data, HIV status, admission discipline (based on primary diagnosis), ward and MRSA screening results were collected from medical records of all screened patients. The costs of the screening program were retrospectively collected from hospital administrative databases where hospital bills were recorded. Interviews with nurse managers of respective wards who oversee the screening program were also done to estimate the staff time spent and the usage of equipment on the various screening activities and procedures. Data from January 2009 to December 2010 were analyzed. Univariate analysis was used to determine associations between demographic or hospital admission with MRSA colonization or acquisition. A chi square test was used to evaluate differences in categorical variables. Crude odds ratios (ORs) and 95 % confidence intervals were calculated for categorical variables. Crude ORs for continuous variables were obtained by using simple logistic regression. Stratified analyses for age (> = 65) was used. All tests were two-tailed, and P values less than 0.05 were considered statistically significant. All variables with a *P* value <0.05 in univariate analysis were included in multivariate analyses. A stepwise logistic regression was used to choose variables that were independently and significantly associated with MRSA colonization or acquisition and for inclusion in the final model. A variable was dropped from the model if it did not reach statistical significance. All data were analyzed using Stata 9 (Stata Corp., College Station, TX).

### Disease economic burden

The estimated disease burden was calculated for patients who were detected to be MRSA positive at admission and progressed to infection. Economic and disease burden include direct and indirect costs. The direct costs are based on hospitalization stay while the indirect costs are represented by the value of productivity loss due to illness-related absence resulting from additional hospitalization [30–32]. The direct costs are estimated as the product of the total average of daily hospital bills of hospital stay due to MRSA colonization per patient classification (i.e. HIV, ID, dermatology), the number of patients per classification and the number of additional hospitalization days per patient (Table 1). The additional hospitalization days was estimated by comparing the length of stay of colonized and non-colonized patients. In addition, we assumed that 35 % of the colonized patients will develop infection [33], and assumed that colonization and infection lead to the same additional hospitalization.

**Table 1:** Direct and indirect costs avoided by the MRSA screening and isolation program

Parameter	Number of colonized patients	Number of Infected patients	Cost per day ($US)	Total cost ($US)
Direct cost				
Hospital bill per day for infectious disease patient	14	5^a^	228	4469^b^
Hospital bill per day for dermatological patient	162	57^a^	372	63,277^c^
Hospital bill per day stay for HIV patient	97	34^a^	226	36,123^d^
Total direct cost				103,869
Indirect cost				
Productivity loss of infectious disease patient	14	5^a^	155^e^	2803
Productivity loss of dermatological patient	162	57^a^	155^e^	24,324
Productivity loss of HIV patient	97	34^a^	155^e^	19,419
Total indirect cost				50,453

^a^Thirty-five percent of the colonized patients are assumed to progress to infection

^b^Based on four additional hospitalization days and five Infectious disease patients estimated to be progressed to infection

^c^Based on three additional hospitalization days and 57Dermatological patients estimated to be progressed to infection

^d^Based on four additional hospitalization days and 34HIV patients estimated to be progressed to infection

^e^Estimated based on GDP per capita of $US 55,183 [36]

Using the human capital approach, the indirect costs are calculated as the product of the gross domestic product per capita per day, the number of patients per classification and the days of work lost due to MRSA colonization (Table 1).

The screening program cost consists of cost of staff needed for swab taking including labeling and preparation, data collection and terminal cleaning of positive rooms, cost of bottles used for hand rubbing, and the screening fees paid by patients. The staff cost was calculated by multiplying staff cost per patient—estimated based on staff time needed and monthly salary—and number of patients. Cost of bottles was calculated by multiplying number of bottles used and cost of one bottle. Screening fees was calculated by multiplying the tariff paid by patient by number of patients (Table 2). All cost estimates were expressed in US$ of 2014 (Table 3).

**Table 2:** Screening program cost in US$^a^

Cost item	HIV patients			Infectious diseases patients			Dermatological patients		
	Staff cost per patient	Number of patients	Total cost	Staff cost per patient	Number of patients	Total cost	Staff cost per patient	Number of patients	Total cost
Swabs (entry)	2.74^b^	913	2504	2.74^b^	520	1426	2.74^b^	824	2260
Cleaning (entry)	2.70^c^	97	262	2.70^c^	14	38	2.70^c^	162	438
Collating (entry)	0.55^d^	913	501	0.55^d^	520	285	0.55^d^	824	452
Swabs (discharge)	2.74^b^	679	1862	2.74^b^	464	1272	2.74^b^	624	1711
Collating (discharge)	0.55^d^	679	371	0.55^d^	464	253	0.55^d^	624	341
Hand rub Bottles		680^e^	5304		104^e^	808		1204^e^	9390
Tariff paid by patient	25	1592	39,736	25	984	24,561	25	1448	36,142
Total (entry and discharge)	50,540			28,643			50,733		

^a^S$ was converted to US$ based on exchange rate of 0.78

^b^Fifteen minutes of staff time is needed to take swabs including labelling/preparation for one patient with a monthly salary of S$2250 [59]

^c^Thirty minutes of staff time is needed to clean MRSA positive room for one patient with a monthly salary of S$950 [59]

^d^Thirty minutes of staff time is needed to collate data for ten patients with a monthly salary of S$2250 [59]

^e^A bottle cost S$10 (~ US$7.8)

**Table 3:** Values of parameters used in DALYs

Parameter	Value
Age weighting correction constant, *C*	0.1658^a^
Social discount rate, *r*	0. 03^a^
Average age of the individuals at the onset of symptoms, *a*	61^b^
Duration of the disability per year, *L*	0.01^c^
Parameter from the age-weighting function, *β*	0.04^a,d^
Disability weight, *D*	0.041^e^

^a^[35]

^b^The Median age for positive MRSA patients reported in Table 4

^c^Calculated by dividing addition hospitalization days (i.e. 4 days) by 356 days

^d^The age weighting function represents the value of life at different ages. It reflects the different social roles of individuals at different ages, i.e. young and elderly require care giving [35]

^e^[60]

The cost-effectiveness analysis was used to determine the cost-per-outcome gained. It considers the direct and indirect costs. Disability-adjusted life years (DALYs) due to MRSA infection were used as an outcome measure for the cost-effectiveness analysis. DALYs were chosen over other metrics as quality-adjusted life year (QALYs) because the WHO recommends that future cost-effectiveness analyses also use DALYs for purposes of comparability [34]. The following equation outlined by Murray [35] was used to calculate the DALYs (Table 1):

1 $$ -\frac{DC{e}^{-\beta a}}{{\left(\beta +r\right)}^2}\left[{e}^{-\left(\beta +r\right)L}\left(1+\left(\beta +r\right)\left(L+a\right)\right)-\left(1+\left(\beta +r\right)a\right)\right] $$

where D is the disability weight; r is the social discount rate; a is the age of the individual at the onset of symptoms; L is the duration of the disability; C is the age-weighting correction constant; and β is the parameter from the age-weighting function. The age weighting function represents the value of life at different ages (Table 3).

Due to lack of information before implementing the program, we used the prevalence of MRSA in the first quarter of our on-going study period (i.e. Jan 2009–March 2009), which was estimated at 2.7 %, as a surrogate for MRSA prevalence if no additional intervention such as de-colonization of patients was taken; and the last quarter of our on-going study period (i.e. April–June 2013), which was estimated at 1.1 %, as a surrogate for MRSA prevalence with the screening program in place. It is assumed that this surrogate could reflect the situation before the program as there is a lag period until the program could have effect on reducing the MRSA prevalence rate. The effectiveness of the program in reducing the MRSA prevalence rate of infected patients was thus assumed to be 1.6 %. The effectiveness was estimated as the difference between the MRSA prevalence of the first (Jan–March 2009) and last quarter (April–June 2013) of our on-going study.

### Ethics approval

This study was approved by Domain Specific Review Board, National Healthcare Group, Singapore (DSRB-2011/01655).

## Results

### Statistical analysis

During the study period, of 2476 patient admissions, 2257 patients were screened for MRSA excluding 115 patient admissions with known MRSA status and 104 patient admissions that refused to do screening. Total number of patient admissions screened from ward A, B and C were 284, 1591 and 382 respectively. Our data show that, of these 104 patient admissions that refused screening on entry, 19 % (20 patients) were screened on discharge.

**Fig. 1:**
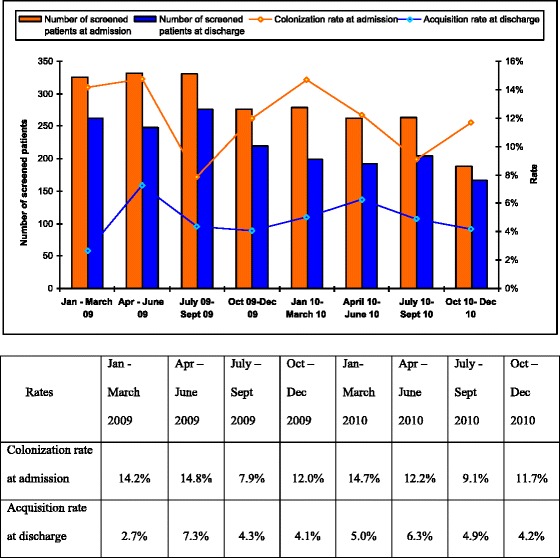
Prevalence and Acquisition of MRSA colonization by quarter during the study period (Jan 2009–Dec 2010). The graph shows the number of screened patients at admission and at discharge, MRSA colonization rate at admission, and MRSA acquisition rate at discharge

Of the 162 MRSA positive screenings from dermatological patients, 21 (13 %) were from wound swabs, compared to only 1 of 111 positive episodes (1 %) among combined ID and HIV patients. The proportion of patients fulfilling the screening criteria decreased as the study period progressed, and the prevalence rate of MRSA colonization fluctuated. Interestingly, colonization was lowest in the July–September quarter during both years. The incidence rate of MRSA acquisition during hospitalization decreased throughout the study period except in the April–June quarter of the second year (Fig. 1). There was a positive correlation between colonization rate at admission and acquisition rate at discharge although it did not reach statistical significance (Spearman’s correlation coefficient 0.38, *p* = 0.352).

The average prevalence rate (excluding patients with known MRSA positive) of screened patients at admission during the study period was 12.1 %. MRSA colonization prevalence rates for ward A, B & C were 12.7, 12.2 and 11.3 % respectively throughout the study period. Univariate analyses of demographic and admission data of all screening episodes are summarized in Table 4. The median age of those who screened positive at admission was 61 versus 46 years for those who screened negative (*p* < 0.001). In those aged 65 years and above, the odds of colonization were 3.91 times greater than those less than 65 years of age (*p* < 0.001). Male patients were less likely to be colonized with MRSA at admission than females (odds ratio [OR], 0.74; 95 % confidence interval [CI], 0.57–0.98; *p* = 0.03).

**Table 4:** Univariate analysis of demographic and admission data of entry screening (*n* = 2257)

	MRSA positive on admission, [*n* = 273 (%)^a^]	MRSA negative on admission, [*n* = 1984 (%)^a^]	OR (95 % CI)	*p* value
Demographics				
Age, median (range)	61 (18–97)	46 (13–109)	1.04 (1.03–1.05)	<0.001
≥ 65 years	118 (43.2)	323 (16.3)	3.91 (2.96–5.16)	<0.001
< 65 years	155 (56.8)	1661 (83.7)	1.0	
Gender				
Male	178 (65.2)	1420 (71.6)	0.74 (0.57–0.98)	0.03
Female	95 (34.8)	564 (28.4)	1.0	
Ethnicity				
Chinese	210 (76.9)	1410 (71.1)	1.36 (1.00–1.86)	0.04
Malay	35 (12.8)	238 (12.0)	1.08 (0.72–1.58)	0.70
Indian	19 (7.0)	181 (9.1)	0.74 (0.43–1.22)	0.24
Others	9 (3.3)	155 (7.8)	0.40 (0.18–0.80)	0.007
Patient groups				
HIV	97 (35.5)	816 (41.1)	0.79 (0.60–1.03)	0.077
Dermatology	162 (59.3)	662 (33.4)	2.91 (2.23–3.81)	<0.001
Infectious disease	14 (5.1)	506 (25.5)	0.16 (0.08–0.27)	<0.001

^a^Unless otherwise indicated

In the univariate analyses, patients colonized with MRSA on admission tended to be Chinese (OR, 1.36; 95 % CI, 1.00–1.86; *p* = 0.04). ID patients were less likely to test positive for MRSA on admission than HIV patients and dermatological patients (OR 0.16; 95 % CI, 0.08–0.27; *p* < 0.001); the opposite was true for dermatological patients (OR 2.91; 95 % CI, 2.23–3.81; *p* < 0.001).

The multivariate analysis showed that patients with older age (adjusted OR [aOR] 1.03; 95 % CI, 1.02–1.04; *p* < 0.001) and dermatological patient status (aOR 1.49; 95 % CI, 1.11–1.20; *p* = 0.008) had higher risk of MRSA colonization at admission, while patients with other infectious diseases (aOR 0.27; 95 % CI, 0.15–0.48; *p* < 0.001) had decreased colonization risk (Table 5).

**Table 5:** Multivariate analysis of independent risk factors associated with MRSA colonization at admission

Risk factor	Coefficient	Adjusted OR (95 % CI)	*p* value
Age (per year of increase)	0.022	1.03 (1.02–1.04)	<0.001
Dermatological patients	0.367	1.49 (1.11–1.20)	0.008
ID patients	−1.325	0.27 (0.15–0.48)	<0.001

Of 1984 patient admissions with MRSA negative on entry, 1767 screenings involving 1357 patients were done at discharge (excluding 130 patient admissions that refused to do screening at exit and 87 patient admissions that discharged/transferred within 48 h after admission). Of these, 85 screening episodes (4.8 %) were MRSA positive. MRSA acquisition rates for ward A, B & C were 8.0, 4.8 and 3.2 % respectively during the study period (Table 6). Patients who acquired MRSA during hospitalization were more likely to be older than those who were negative (median age 55 vs. 46 years, respectively; *p* < 0.001). Gender and ethnicity were not statistically significant factors for MRSA acquisition.

**Table 6:** Univariate analysis of demographic and admission data on acquisition (*n* = 1767)

	Acquired MRSA, [*n* = 85 (%)^a^]	Did not acquire MRSA, [*n* = 1682 (%)^a^]	OR (95 % CI)	*p* value
Demographics				
Age, median (range)	55 (15–109)	46 (13–109)	1.03 (1.01–1.04)	<0.001
≥ 65 years	28 (32.9)	273 (16.2)	2.53 (1.52–4.14)	<0.001
< 65 years	57 (67.1)	1409 (83.8)	1.0	
Gender				
Male	59 (69.4)	1180 (70.2)	0.96 (0.59–1.62)	0.88
Female	26 (30.6)	502 (29.8)	1.0	
Ethnicity				
Chinese	63 (74.1)	1197 (71.2)	1.2 (0.69–2.00)	0.56
Malay	9 (10.6)	202 (12.0)	0.87 (0.38–1.77)	0.69
Indian	7 (8.2)	157 (9.3)	0.87 (0.33–1.93)	0.73
Others	6 (7.1)	126 (7.5)	0.94 (0.33–2.19)	0.88
Patient groups				
HIV	32 (37.7)	647 (38.5)	0.97 (0.60–1.54)	0.880
Dermatology	46 (54.1)	578 (34.4)	2.25 (1.42–5.39)	0.0002
Infectious disease	7 (8.2)	457 (27.2)	0.24 (0.09–0.53)	0.0001
Admission ward				
Single-bed room	61 (71.8)	1223 (72.7)	0.95 (0.58–1.62)	0.849
Multi-bed room	24 (28.2)	459 (27.3)	1.0	
Length of stay, days; median (range)	8 (2–105)	5 (1–145)	1.04 (1.02–1.06)	<0.001

^a^ Unless otherwise indicated

The duration of hospital stay was longer for patients who acquired MRSA compared to patients who did not (OR 1.04; 95 % CI, 1.02–1.06; *p* < 0.001). ID patients were less likely to acquire MRSA than HIV patients and dermatological patients (OR 0.24; 95 % CI, 0.09–0.53; *p* = 0.0001). Conversely, MRSA acquisition was more likely to occur in dermatological patients compared to HIV patients and ID patients (OR 2.25; 95 % CI, 1.42–5.39; *p* = 0.0002). MRSA acquisition was not associated with ward layout (OR 0.95; 95 % CI, 0.58–1.62).

The multivariate analysis showed that age (aOR 1.02; 95 % CI, 1.01–1.03; *p* = 0.002) and length of stay (aOR 1.04; 95 % CI, 1.03–1.06; *p* < 0.001) were predictors of MRSA acquisition. Compared to dermatological and HIV patients, ID patients were less likely to acquire MRSA during hospital stay (aOR, 0.25; 95 % CI, 0.11–0.60; *p* = 0.002) (Table 7).

**Table 7:** Multivariate analysis of independent risk factors associated with MRSA acquisition

Risk factor	Coefficient	Adjusted OR (95 % CI)	*p* value
Age (per year of increase)	0.016	1.02 (1.01–1.03)	0.002
Length of stay (per day of increase)	0.042	1.04 (1.03–1.06)	<0.001
ID patients	−1.208	0.25 (0.11–0.60)	0.002

### Economic and disease burden

For the study period 2009–2010, the total hospitalization direct cost for all patients was estimated to be $103,869. This cost was divided between hospitalization cost of $4469 for ID patients, $63,277 for dermatological patients, and $36,123 for HIV patients, based on hospitalization of four days for HIV and ID patients and three days for dermatological patients with MRSA acquisition. On average, the daily hospital bill of dermatological patients ($372) was higher than that of the ID ($228) and HIV patients ($266). The total value of productivity loss for MRSA patients was estimated at $50,453. The productivity loss for HIV, dermatological and ID patients was $21,049, $26,366 and $3038, respectively.

The total cost of the program was $129,916. Costs of cleaning positive rooms, collating data, swabbing and hand rub bottles used were estimated at $738, $2203, $11,035 and $15,502 respectively. The screening cost paid per patient is $25, leading to a total screening cost of $100,439.

By weighing the costs of the screening program against the direct costs that can be avoided as a result of implementing the program, the results show that the program costs are 1.3 times higher than the avoided direct costs, while if we weigh the program costs against the avoided direct and indirect costs, we will find that the avoided total costs are 1.1 times higher than the program costs. The cost-effectiveness ratio (CER) was estimated to be −400 $/DALY averted (and −428 $ per infection prevented), and estimated at −197, 3949, and −663 $/DALY averted for HIV, ID and dermatological patients, respectively. Using the Singaporean per capita gross national income of $55,183 as the cost-effectiveness threshold [36], we find that the isolation and screening program is cost saving as it is less than this threshold and with a negative value, implying that the interventional program is not only economically justified but gains could also be expected from implementing this program.

### Uncertainty analysis

In order to investigate the robustness of the estimated impacts and the effectiveness of the screening program, Monte-Carlo simulations (1000 iterations) were conducted to account for uncertainty in the percentage of colonized patients who develop infection (triangular distribution of 0.11, 0.35, 0.60) [37–39], the disability weights used in DALYs calculation (i.e. triangular distribution of 0.041, 0.07, 0.65), and the estimated effectiveness of the program which is determined from the selected duration of our on-going study (i.e. triangular distribution of one, two and three quarters).

**Fig. 2:**
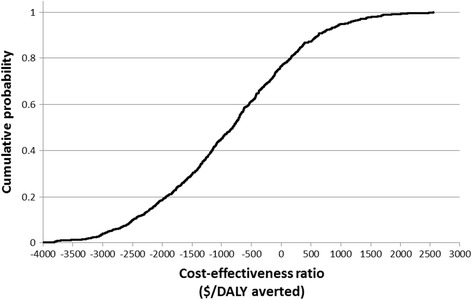
Cumulative distribution of the cost-effectiveness of the screening program. The results showed that the estimated mean and standard deviation of the cost-effectiveness were −198 and 148 $/DALY averted, while the 5th and 95th percentiles were estimated at −441 and 15 $/DALY averted

The results showed that the estimated mean and standard deviation of the cost-effectiveness were −198 and 148 $/DALY averted, while the 5th and 95th percentiles were estimated at −441 and 15 $/DALY averted (Fig. 2).

## Discussion

This study is different from previously published studies because it took place in a unique setting which houses only patients with infectious diseases and/or dermatology inpatients. The screening program was rolled out with the administrative decision of screening only HIV patients in ward A as the length of stay for general infectious disease cases was short with an average of 2–4 days. Screening of HIV patients with longer hospital stay allowed studying the epidemiology and risk factors for MRSA acquisition in this specific patient population.

A previous study done at two general hospitals in Singapore by Pada et al. [27] found that inpatient cases with clinically significant healthcare-associated MRSA infections were less likely to be of Chinese ethnicity compared to non-infected controls. However, the reverse is true for colonization as we found in this study that among different ethnic groups, patients with Chinese origin were more likely to have colonized with MRSA upon screening on admission than patients of non-Chinese origins in univariate analysis. Further study is required to verify the previous finding in our setting.

We also found that elderly patients and dermatological patients had a higher prevalence of MRSA colonization. Our findings are consistent with other studies which showed that patients with skin lesions and elderly patients had higher chances of MRSA carriage on hospital admission [10, 28]. It is not impossible that the high prevalence of MRSA colonization in dermatological patients in this study reflects community-associated (CA-MRSA) or that those with skin lesions are at higher risk of contracting MRSA. We noted studies in Singapore reported increasing trend of CA-MRSA cases in Singapore [40, 41].

It has been shown that the presence of chronic skin breaks is significantly associated with MRSA acquisition [42, 43]. In this study, we do not know if all patients admitted for skin diseases had chronic skin breaks. In order to explain why ID patients were less likely to screen positive at admission, we observed that dermatological patients were older than ID patients (median age 72 vs. 49 years, respectively).

We also found that longer hospital length of stay was an independent predictor of MRSA acquisition. This result confirmed previous findings where length of stay was a predictor of MRSA acquisition both in the general wards and the intensive care unit [44–46]. This may be an indicator that patients with more severe underlying diseases may require longer stays in the hospital, and these long stayers likely need increased nursing care requirements. It has been shown that multiresistant micro-organisms have been isolated from the hands, gloves, aprons, and other instruments used by healthcare workers engaged in the care of patients who are colonized or infected by these organisms [47]. Though there is not likely to be a single solution for MRSA control, hand hygiene is vital and the most basic measure among several elements to help prevent the spread of MRSA. Despite recent infection control activities such as hand hygiene campaigns and implementation of hand hygiene audits [48], the finding that MRSA acquisition at CDC does occur highlights the need to review nursing workloads, adequate staffing resources, and adherence to infection control practices.

This study is the first to our knowledge that evaluates the cost-effectiveness of screening and isolation of MRSA patients in a tropical country. Another unique feature of the analysis is the evaluation of acquisition rates among specific types of patients (dermatological, HIV and infectious disease patients) and the comparison of the cost-effectiveness of screening and isolation between them, showing that marked differences exist between patient types. This is due to a combination of risk factors and length of hospital stay that affect the cost and disease profile of the patients.

Our results point towards high MRSA prevalence that can be cost-effectively reduced by selective screening and isolation programs in pre-selected subpopulations in Singapore. On average the CER was estimated at −400 $/DALY averted. The screening program was cost saving for HIV patients (−197 $/DALY), dermatological patients (−663 $/DALY) and cost effective for ID patients (3949 $/DALY), respectively. Our findings thus demonstrate that screening and isolation of patients can be highly cost-effective in a tropical setting. These findings are specific to the high indirect costs associated to job absenteeism and high hospitalization costs in Singapore. These results, nonetheless, would offer insights in other tropical countries that are increasingly approaching high-income status such as Brazil or Malaysia and which presumably would present similar prevalence levels for MRSA. Our results could also be extrapolated to other specialized hospital settings dealing with HIV and dermatological patients, offering insights on isolation and screening strategies.

Pereira et al. examined MRSA control activities in Singapore and found that isolation policies and screening practices were inconsistent among health care institutions [49]. Universal screening coupled with isolation and decolonization of patients has been identified as the most effective interventions in mathematical models [50]. In our setting, MRSA-positive patients might have been a potential source of spread if we did not practice isolation for those who tested positive during initial screening.

Studies assuring the effectiveness of universal screening in decreasing MRSA prevalence showed that there was a substantial reduction in MRSA infections with screening and decolonization [6, 51]. Nonetheless, resource implication would be an issue for universal screening with isolation and decolonization. As such, selective screening strategies could detect colonized patients who are at risk to reduce the current prevalent and acquisition rates in hospitals, and it could spare other patients from the burden of serious infection. Active MRSA selective screening followed by appropriate infection control practices has been shown to be successful in reducing acquisition rates in hospitals. Such strategies have been incorporated into many institutionalized MRSA control programs [52–54]. The effectiveness of isolation measures in reducing the incidence of MRSA colonization and infection in hospital inpatients is however not yet quantitatively strong, due to lack of consideration of confounders and appropriate statistical analyses [55]. In addition, there is not yet consensus about the optimal screening strategy and the course of action once MRSA infected patients are detected [56], ranging from routine decolonization of all carriers in Denmark and the Netherlands to only particularly transmissible or virulent MRSA clones being decolonized in Western Australia [57].

In the present study, MRSA carriage rate on hospital admission in this patient population was 12 %. This high rate of MRSA colonization is alarming because the chances of developing an invasive infection are high once a patient is colonized [33, 38, 58].

There are limitations to our study. Our findings may not be nationally representative as the screening program was done only in one institution that houses patients with specific medical conditions. Our screening policy needs the capacity to strictly isolate or cohort patients which might not be feasible at a setting where there is limited availability of isolation facilities. We also acknowledge that in our study, 4 % of patient admissions with unknown MRSA history were not screened on admission and this will affect MRSA colonization pressure in the ward. Our hospital started hand hygiene audits in the year of 2010, thus we failed to include this in our current study. Additionally, we did not look at co-morbidities or other risk factors for MRSA colonization or acquisition such as health seeking behavior, previous hospitalization history and recent antibiotic usage as the data reported here were gathered for operational purposes. Moreover, regarding the estimation of economic burden of the disease, we could not account for some costs such as microbiology lab material and additional costs to isolate detected MRSA patients who would have otherwise remained in less costly multiple bed rooms. Missing information on the prevalence of MRSA before implementing the screening program and using instead the first quarter of our study as a surrogate for the baseline without the screening program was a limiting factor in our CEA. The robustness of this assumption was considered in the uncertainty analysis in the estimated effectiveness rate of the program by varying the selected duration to from one to two and three quarters, showing that our results are robust to this assumption.

Despite these limitations, a number of practical issues were identified. High prevalence and acquisition of MRSA within this center reinforces the need for MRSA prevention and control, and increased hand hygiene compliance. Resource utilization would be an issue for universal screening or sustaining selected screening of MRSA in the long run. However, this study demonstrated the cost-effectiveness of selected screening and isolation, which strongly favors the continuation of such efforts. More clinical research is needed to understand the risk factors for MRSA acquisition during hospitalization and the utility of molecular epidemiology of MRSA to study the spread of MRSA, including the interplay between hospital and community MRSA.

## Conclusions

Overall our results indicate high MRSA prevalence that can be cost effectively reduced by selectivescreening and isolation programs in Singapore.
